# Genotype Variation in Rice (*Oryza sativa* L.) Tolerance to Fe Toxicity Might Be Linked to Root Cell Wall Lignification

**DOI:** 10.3389/fpls.2019.00746

**Published:** 2019-06-12

**Authors:** Ricardo José Stein, Guilherme Leitão Duarte, Lívia Scheunemann, Marta Gomes Spohr, Artur Teixeira de Araújo Júnior, Felipe Klein Ricachenevsky, Luis Mauro Gonçalves Rosa, Nilson Ivo Tonin Zanchin, Rinaldo Pires dos Santos, Janette Palma Fett

**Affiliations:** ^1^Faculdade Murialdo, Caxias do Sul, Brazil; ^2^Centro de Biotecnologia, Universidade Federal do Rio Grande do Sul, Porto Alegre, Brazil; ^3^Instituto de Biociências, Universidade Federal do Rio Grande do Sul, Porto Alegre, Brazil; ^4^Departamento de Biologia, Universidade Federal de Santa Maria, Santa Maria, Brazil; ^5^Departamento de Plantas Forrageiras e Agrometeorologia, Faculdade de Agronomia, Universidade Federal do Rio Grande do Sul, Porto Alegre, Brazil; ^6^Instituto Carlos Chagas, Fundação Oswaldo Cruz – FIOCRUZ, Curitiba, Brazil

**Keywords:** iron, rice, lignin, root, exclusion

## Abstract

Iron (Fe) is an essential element to plants, but can be harmful if accumulated to toxic concentrations. Fe toxicity can be a major nutritional disorder in rice (*Oryza sativa*) when cultivated under waterlogged conditions, as a result of excessive Fe solubilization of in the soil. However, little is known about the basis of Fe toxicity and tolerance at both physiological and molecular level. To identify mechanisms and potential candidate genes for Fe tolerance in rice, we comparatively analyzed the effects of excess Fe on two cultivars with distinct tolerance to Fe toxicity, EPAGRI 108 (tolerant) and BR-IRGA 409 (susceptible). After excess Fe treatment, BR-IRGA 409 plants showed reduced biomass and photosynthetic parameters, compared to EPAGRI 108. EPAGRI 108 plants accumulated lower amounts of Fe in both shoots and roots compared to BR-IRGA 409. We conducted transcriptomic analyses of roots from susceptible and tolerant plants under control and excess Fe conditions. We found 423 up-regulated and 92 down-regulated genes in the susceptible cultivar, and 42 up-regulated and 305 down-regulated genes in the tolerant one. We observed striking differences in root gene expression profiles following exposure to excess Fe: the two cultivars showed no genes regulated in the same way (up or down in both), and 264 genes were oppositely regulated in both cultivars. Plants from the susceptible cultivar showed down-regulation of known Fe uptake-related genes, indicating that plants are actively decreasing Fe acquisition. On the other hand, plants from the tolerant cultivar showed up-regulation of genes involved in root cell wall biosynthesis and lignification. We confirmed that the tolerant cultivar has increased lignification in the outer layers of the cortex and in the vascular bundle compared to the susceptible cultivar, suggesting that the capacity to avoid excessive Fe uptake could rely in root cell wall remodeling. Moreover, we showed that increased lignin concentrations in roots might be linked to Fe tolerance in other rice cultivars, suggesting that a similar mechanism might operate in multiple genotypes. Our results indicate that changes in root cell wall and Fe permeability might be related to Fe toxicity tolerance in rice natural variation.

## Introduction

Iron (Fe) is an essential nutrient for plants. It is involved in oxi-reductive reactions in photosynthesis, respiration and nitrogen assimilation, as well as in other important plant biological processes. Thus, plants have evolved mechanisms to maintain Fe homeostasis when soil concentration is low (Sperotto et al., 2012; Ricachenevsky et al., 2018). However, Fe can also become toxic if accumulated inside the cell, acting as a potent generator of reactive oxygen species (ROS), specially the hydroxyl radical, by the Fenton reaction (Becana et al., 1998). This radical is extremely toxic to cell metabolism, leading to oxidation of biological macromolecules such as lipids, proteins and nucleic acids, causing membrane leakage and even cell death (Blokhina et al., 2003). Thus, plants must maintain Fe concentrations within a narrow range for proper growth and development.

Rice plants are especially prone to Fe toxicity when cultivated under flooded conditions. Well-aerated soils usually have high amounts of ferric Fe (Fe^3+^), which has low solubility. Waterlogging, however, results in an anoxic and reductive environment that reduces Fe^3+^ to the more soluble Fe^2+^, which is accumulated in the soil solution (Becker and Asch, 2005). Wetland rice stands for most of the world rice production, and Fe toxicity reduces rice yield by 12 to 100%, depending on the genotype, intensity of Fe toxicity stress and soil fertility status (Sahrawat, 2004). Two distinct types of toxicity have been described in the literature: a true (or real) Fe toxicity – characterized by the accumulation of toxic levels of Fe in the plant body (Sahrawat, 2000; Olaleye et al., 2001; Stein et al., 2009a) and an indirect toxicity, caused by Fe precipitation in the root apoplast – the Fe plaque – resulting in multiple nutritional deficiencies (Sahrawat, 2004).

Different Fe toxicity tolerance mechanisms have been proposed for rice plants: type I consists in Fe exclusion from roots, and uses aerenchyma-derived oxygen or enzymatic activity to oxidize Fe^2+^ into Fe^3+^, which precipitates as an Fe plaque at the root surface (Wu et al., 2014); type II consists of shoot tolerance to high Fe concentration, likely through compartmentalization via storage within the inner cavity of ferritin proteins (Stein et al., 2009b) or by the action of vacuolar transporters such as VITs (VACUOLAR IRON TRANSPORTER, Zhang et al., 2012); and type III, in the presence of an antioxidant system that detoxifies reactive oxygen species produced via Fenton when Fe is present in excess (Wu et al., 2017). Type I is considered a root-based mechanism, and types II and III are shoot-based mechanisms. Clearly, there is wide variation in tolerance, which depends on the stress duration, strength, and plant developmental stage. Some genotypes may present contrasting phenotypes depending on how the experiments are performed (Wu et al., 2014; Matthus et al., 2015), indicating the need to better understand the underlying physiological and molecular mechanisms of Fe tolerance. Previous work showed that (1) photosynthesis is affected by Fe toxicity (Stein et al., 2009a; Müller et al., 2017); known Fe uptake genes are down-regulated upon high Fe treatment (Finatto et al., 2015); and that early (3 days) and late (3 weeks) Fe toxicity responses are quite different in both roots and shoots (Quinet et al., 2012). However, molecular mechanisms associated with tolerant and sensitive genotypes are underexplored.

Although no causative gene that confers Fe tolerance was cloned, previous studies showed that Fe tolerance in distinct rice genotypes is a quantitative trait, with many QTL already described, all showing small effects (Dufey et al., 2009, 2012, 2015; Wu et al., 2014; Matthus et al., 2015; Zhang et al., 2017). Based on these studies, shoot-based tolerance might be linked to two glutathione S-transferases localized in chromosome 1, which are induced upon Fe stress (Matthus et al., 2015). For root-based mechanisms of Fe exclusion, one QTL co-localizes with OsIRT1, which is a Fe^2+^ transporter involved in the combined strategy for Fe acquisition present in rice (Ishimaru et al., 2006; Sperotto et al., 2012; Ricachenevsky and Sperotto, 2014).

Here, we analyzed the effects of high Fe concentration on two rice cultivars previously characterized as Fe toxicity susceptible and tolerant, BR-IRGA 409 and EPAGRI 108 (Silveira et al., 2007). The two cultivars are commonly used in rice fields in the two southernmost provinces of Brazil, Rio Grande do Sul, and Santa Catarina. Besides physiological characterization, transcriptomic analyses using microarrays were performed, showing markedly different gene expression profiles in the two contrasting cultivars. Our data suggest that tolerance to high Fe in some rice cultivars can be linked to increased lignification, which would in turn result in decreased permeability for Fe radial diffusion in roots and consequently lower root-to-shoot Fe translocation.

## Results

### BR-IRGA 409 and EPAGRI 108 Susceptibility and Tolerance to Fe Toxicity

**Figure 1:**
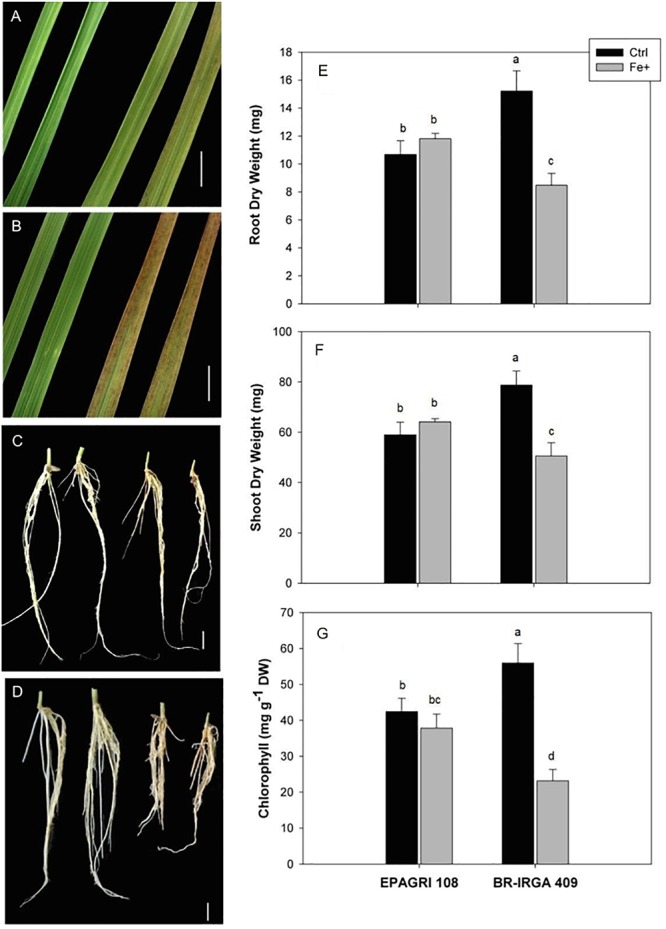
Effects of excess iron in leaves **(A,B)** and roots **(C,D)** from EPAGRI 108 **(A,C)** and BR-IRGA 409 **(B,D)** rice plants exposed for 9 days to control (left) and excess (right) iron treatments. Bars represent 0.5 cm. Root **(E)**, and shoot **(F)** dry weight, and total chlorophyll **(G)** of plants exposed for 9 days to control (Ctrl) or iron excess (Fe+). Each value represents the mean of six replicates ± SE. Distinct letters above the bars indicate significant difference between means (Duncan test, *P* ≤ 0.05).

BR-IRGA 409 and EPAGRI 108 (hereafter “susceptible” and “tolerant” cultivars) were previously characterized as susceptible and tolerant to Fe toxicity, respectively, based solely on shoot biomass (Silveira et al., 2007). In order to further characterize the physiological responses of these cultivars to high Fe, we exposed plants to 500 ppm (excess Fe) and 6.5 ppm (control) for 9 days. Plants from the susceptible cultivar developed typical symptoms of Fe toxicity, with appearance of bronzing and necrotic lesions on leaves, while roots turned brown/orange (Figure 1B,D). The tolerant cultivar showed weaker symptoms of Fe toxicity in both shoots and roots (Figure 1A,C). Confirming previous data, shoot and root dry weight of the susceptible cultivar were decreased upon excess Fe treatment (Figure 1E,F), while no significant differences were observed in shoot and root dry weight in the tolerant cultivar. Exposure to excess Fe also led to a severe reduction of chlorophyll concentration in the susceptible cultivar, but no reduction in the tolerant one (Figure 1G). This clearly shows that BR-IRGA 409 and EPAGRI 108 are susceptible and tolerant to excessive Fe in our experimental conditions.

In order to find possible differences in Fe partitioning that could account for differences in tolerance, we quantified Fe concentrations in plants from both cultivars under control and excess Fe conditions (Table 1). Interestingly, shoots and roots (after Fe plaque removal – see below) from both cultivars showed comparable concentrations of Fe. Upon excess Fe treatment, shoots, and roots of the susceptible cultivar increased Fe concentration to a largest extent compared to the tolerant one. In shoots the susceptible cultivar had an increase of sevenfold, whereas the tolerant one had a fourfold increase (Table 1). In order to account for the larger biomass of the tolerant cultivar (Figure 1), we also considered the total Fe content (Fe concentrations in roots and shoots multiplied by the respective dry weights) in both cultivars. The average total Fe contents within plants (roots plus shoots, excluding the “Fe plaque”) under the control treatment were 27.8 and 35.3 mg of Fe in EPAGRI 108 and BR-IRGA 409 plants, respectively. Upon excess Fe treatment, those contents reached 100.8 μg Fe in the tolerant cultivar (EPAGRI 108) and 129.4 μg Fe in the susceptible one. Therefore, even with lower biomass, total Fe uptake in plants from the susceptible cultivar was 28% higher than in the tolerant plants. Similarly, the Fe concentration in roots “Fe plaque” was higher in plants submitted to excess Fe treatment than in control plants, again to a larger extent in the susceptible cultivar. Interestingly, root Fe concentration in the “Fe plaque” was lower under excess Fe conditions in the tolerant cultivar than in the susceptible one (Table 1). Taken together, these results indicate that EPAGRI 108 is capable of excluding Fe from entering the root symplast, thus decreasing root to shoot Fe translocation.

**Table 1:** Iron accumulation and distribution in rice plants from cultivars EPAGRI 108 and BR-IRGA 409 after 9 days of exposure to control (Control) or excess (Fe+) iron treatments.

	EPAGRI 108		BR-IRGA 409	
	Control	Fe+	Control	Fe+
Shoot (mg Fe g^-1^ DW)	0.30 ± 0.02c	1.25 ± 0.11b	0.29 ± 0.03c	2.04 ± 0.21a
Root (mg Fe g^-1^ DW)	0.91 ± 0.16c	1.97 ± 0.23b	0.81 ± 0.15c	2.58 ± 0.2a
Iron plaque (mg Fe g^-1^ DW)	48.07 ± 0.94d	88.81 ± 2.53b	39.04 ± 2.58c	104 ± 2.65a

*Each value represents the mean of six replicates. Distinct letters indicate significant difference between means from each parameter (*P* ≤ 0.05)*.

### Gas Exchange Measurements Reflect the Susceptible and Tolerant Phenotypes

**Figure 2:**
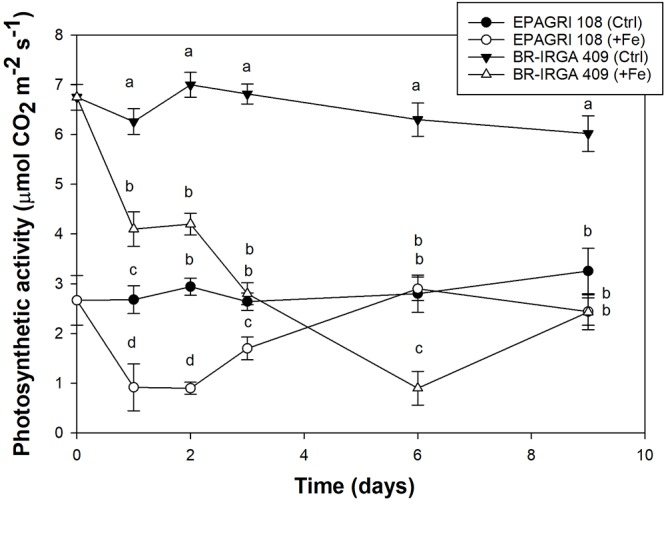
Net CO_2_ assimilation rate from cultivars BR-IRGA 409 (circles) and EPAGRI 108 (triangles) rice plants, exposed to control (closed symbols) or excess (open symbols) iron treatments. Gas exchange measurements were performed after 1, 2, 3, 6,and 9 days of exposure to treatments, using the youngest fully expanded leaf from each plant. Each value represents the mean of six replicates ± SE.

Plants of both cultivars were exposed to excess Fe and control conditions for gas exchange measurements. Plants from the susceptible cultivar exposed to excess Fe showed lower rates of light saturated photosynthesis compared to control plants, while plants from the tolerant cultivar showed little difference between treatments (Figure 2). Decrease on carbon assimilation in BR-IRGA 409 plants was detected as early as the first day of exposure to excess Fe, with further decrease during the 9 days of treatment. In EPAGRI 108, a similar decrease in carbon assimilation was observed after 1 day of treatment. However, after 6 days of excess Fe treatment, plants from the tolerant cultivar recovered, and the photosynthetic activity reached levels similar to those of the control treatment. Thus, the tolerant cultivar is able to circumvent the effects of Fe toxicity to maintain carbon assimilation.

**Figure 3:**
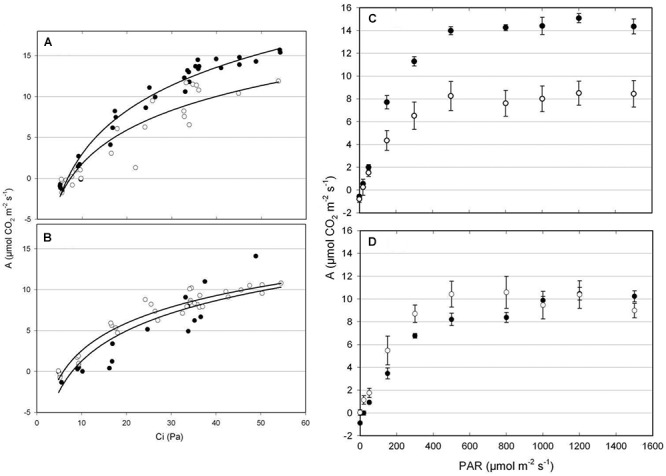
CO_2_ response **(A,B)** and light response **(C,D)** curves from BR-IRGA 409 **(A,C)** and EPAGRI 108 **(B,D)** rice plants after 6 days of exposure to control (closed circles) or excess (open circles) iron treatments. Photosynthetic response curves were obtained for the youngest fully expanded leaf from each plant. Each curve represents the measurements obtained from four independent plants. In C and D, means ± SE are shown. A, net CO_2_ assimilation rate; Ci, estimated substomatal CO_2_ partial pressure; PAR, photosynthetic active radiation.

Different patterns of CO_2_ assimilation rates/estimated substomatal CO_2_ partial pressure (A/Ci) response curves were observed in tolerant EPAGRI 108 and susceptible BR-IRGA 409 plants (Figure 3A,B). Under excess Fe, plants from both cultivars showed a reduction in photosynthetic capacity (the maximum rate of photosynthesis reached under CO_2_ saturation as light is already saturating), which was clearer in BR-IRGA 409 plants. Plants from the susceptible cultivar also showed a decrease in the slope of the A/Ci relationship, indicating a reduction in the carboxylation efficiency under excess Fe compared to control conditions (Figure 3A). Plants from the tolerant cultivar, however, showed no reduction (Figure 3B). Analysis of the response curves by using the model proposed by Farquhar et al. (1980) indicated that excess Fe led to a decrease in maximum carboxylation rate (Vc_max_, 24.5% reduction) and electron transport rate (J_max_, 41.7% reduction) after 6 days of exposure only in the susceptible cultivar (BR-IRGA 409), compared to the control treatment. EPAGRI 108 plants showed no statistically significant changes in both parameters (Table 2).

**Table 2:** Effects of 6-days exposure to control (Control) or excess (Fe+) iron treatments on photosynthetic parameters of rice plants from cultivars EPAGRI 108 and BR-IRGA 409.

	EPAGRI 108		BR-IRGA 409	
	Control	Fe+	Control	Fe+
Vc_max_ (μmol CO_2_ m^-2^s^-1^)	17.75 ± 2.05c	18.65 ± 1.74c	30.78 ± 0.7a	23.25 ± 2.30b
J_max_ (μmol m^-2^ s^-1^)	58.3 ± 15.27b	65.6 ± 12.52b	111.55 ± 12.59a	65.05 ± 5.73b
ϕ_m_ (mol CO_2_ mol^-1^ photons)	0.0369 ± 0.0059b	0.0394 ± 0.0044b	0.0524 ± 0.0043a	0.0252 ± 0.0056c

*Each value represents the mean of four replicates. Distinct letters indicate significant difference between means from each parameter (*P* ≤ 0.05). ϕ_*m*_, apparent quantum yield; Vc_*max*_, maximum carboxylation rate; J_*max*_, electron transport rate*.

The light response curves (Figure 3C,D) revealed a clear advantage for the susceptible BR-IRGA 409 plants under non-stressful conditions, while tolerant EPAGRI 108 plants showed no differences when cultivated under control or excess Fe treatments,. Exposure of BR-IRGA 409 plants to excess Fe resulted in a significant reduction (51.9%) on the apparent quantum yield (ϕ_m_) as compared to control plants, while a small reduction (6.3%, not significant) was observed in EPAGRI 108 plants (Table 2). These data indicate that photosynthesis is only marginally affected by excess Fe in plants from the tolerant cultivar, whereas photosynthetic parameters showed significant decreases in plants from the susceptible one when exposed to excess Fe.

### Oxidative Metabolism Is Affected in Leaves of the Susceptible but Not in Tolerant Cultivar Under Excess Fe

**Figure 4:**
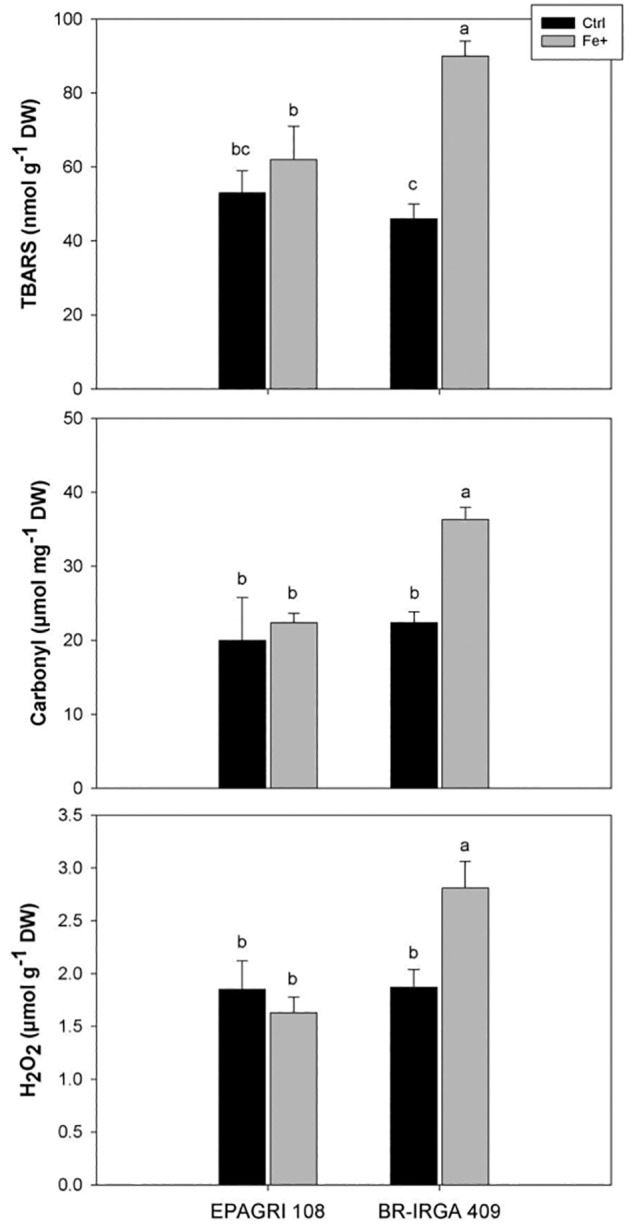
Oxidative damage to lipids and proteins (TBARS and Carbonyl accumulation, respectively) and H_2_O_2_ accumulation in fully expanded leaves from EPAGRI 108 and BR-IRGA 409 rice plants after 9 days of exposure to control (Ctrl) or excess (Fe+) iron treatments. Each value represents the mean of six replicates ± SE. Distinct letters above the bars indicate significant difference between means (Duncan test, *P* ≤ 0.05).

Differences in oxidative metabolism were already suggested as possible sources of tolerance to Fe toxicity in rice (Wu et al., 2017). In the present work, fully expanded leaves from plants of the susceptible cultivar exposed to excess Fe showed higher thiobarbituric acid-reacting substances (TBARS; Figure 4A), carbonyl concentration (Figure 4B) and H_2_O_2_ levels (Figure 4C) than leaves from plants in control conditions, while no difference could be observed in leaves of the tolerant cultivar (Figure 4). These data clearly indicated that excess Fe caused oxidative stress only in the susceptible cultivar (BR-IRGA 409), increasing to oxidation of lipids, proteins and the H_2_O_2_ accumulation in the leaves, whereas no changes are observed in the tolerant cultivar.

**Figure 5:**
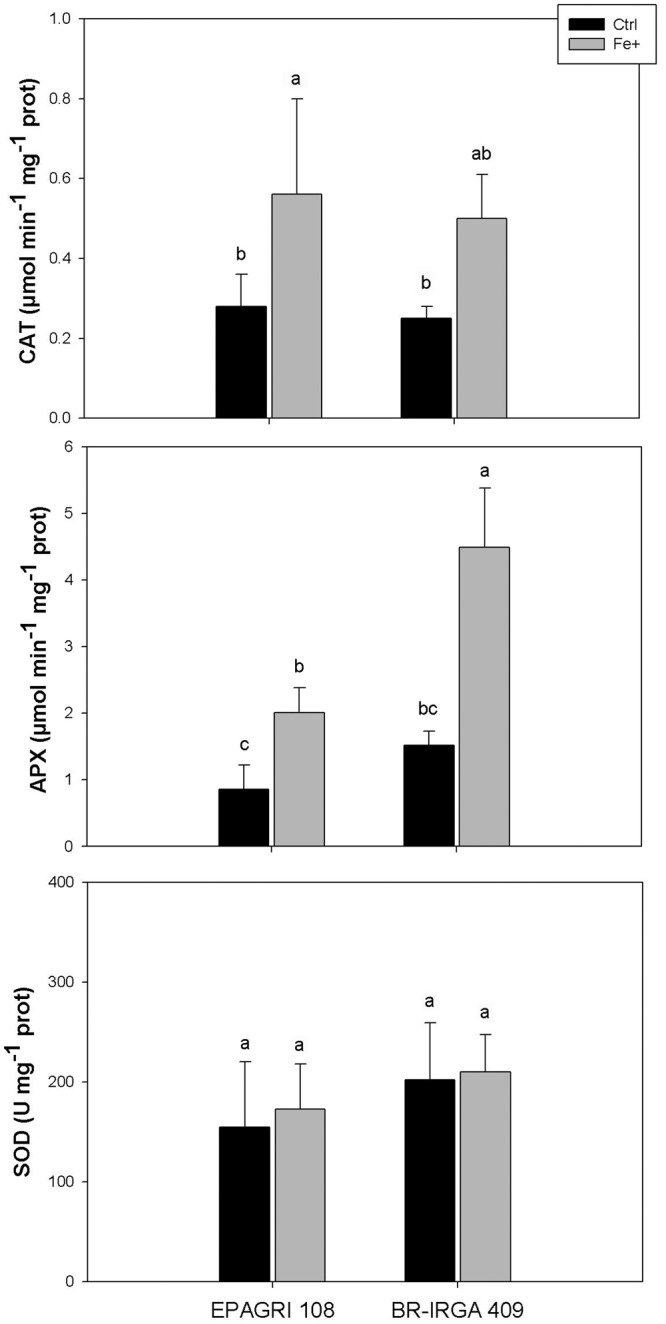
Activity of antioxidant enzymes (CAT, APX, and SOD) in fully expanded leaves from EPAGRI 108 and BR-IRGA 409 rice plants after 9 days exposure to control (Ctrl) or excess (Fe+) iron treatments. Each value represents the mean of six replicates ± SE. Distinct letters above the bars indicate significant difference between means (Duncan test, *P* ≤ 0.05).

Antioxidative enzyme activities were differentially regulated upon excess Fe in each cultivar. Compared to controls, catalase (CAT) activity was increased by Fe toxicity in both cultivars, whereas ascorbate peroxidase (APX) activity was increased to a higher extent in the susceptible one (Figure 5A,B). No difference in superoxide dismutase (SOD) activity was observed in plants from both cultivars submitted to excess Fe compared to controls (Figure 5C). Although we cannot rule out a shoot-based mechanism for EPAGRI 108 Fe tolerance (e.g., increased Fe compartmentalization in vacuoles), these data indicate that the tolerant cultivar has few changes in the antioxidant metabolism that could account for the tolerant phenotype. Thus, EPAGRI 108 seems to be at least to some extent tolerant to excessive Fe due to root-based mechanisms.

### The Gene Expression Profiles of Plants Exposed to Excess Fe Are Highly Divergent

**Figure 6:**
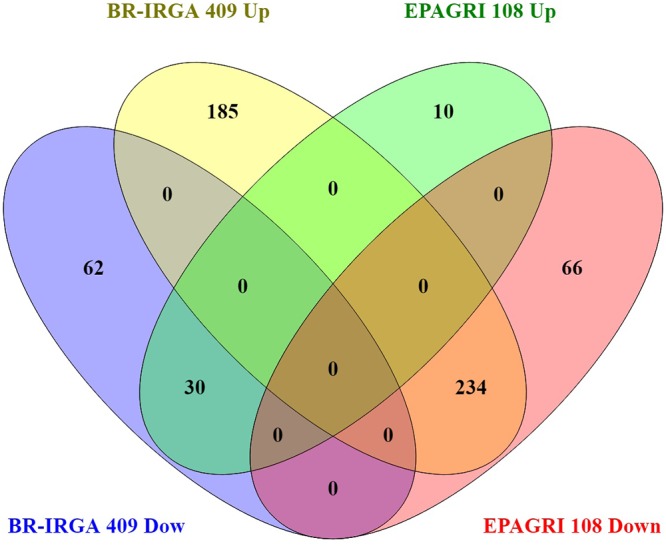
Venn diagram showing overlap between BR-IRGA 409 up-regulated genes, BR-IRGA 409 down-regulated genes, EPAGRI 108 up-regulated genes, and EPAGRI 108 down-regulated genes.

**Figure 7:**
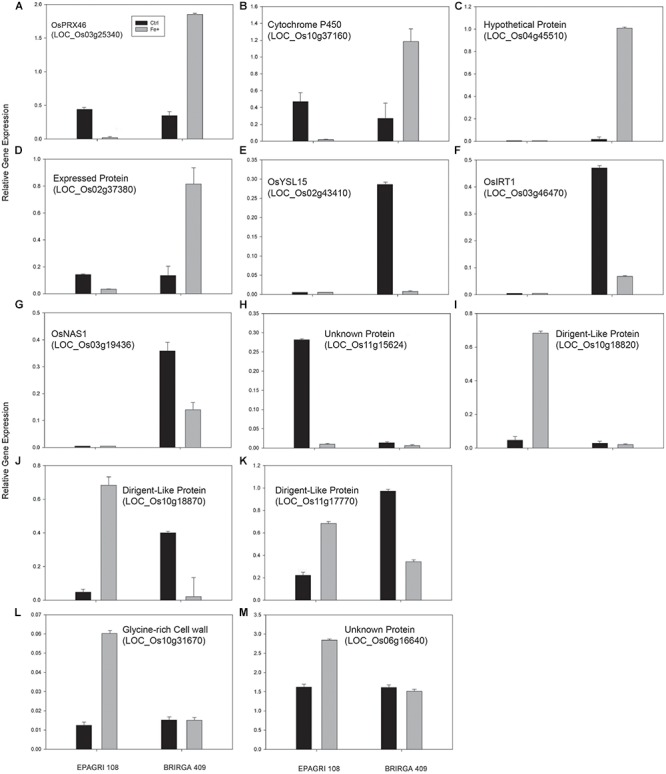
Validation of the microarray analysis by real-time PCR. Relative expression levels by RT-qPCR of selected genes were evaluated in roots of rice plants exposed for 3 days to control or excess iron treatments. **(A)** OsPRX46; **(B)** Cytochrome P450; **(C)** Hypothetical protein; **(D)** Expressed protein; **(E)** OsYSL15; **(F)** OsIRT1; **(G)** OsNAS1; **(H)** Unknown protein; **(I)** Dirigent-Like Protein; **(J)** Dirigent-Like Protein; **(K)** Dirigent-Like Protein; **(L)** GLycine-rich Cell wall protein; **(M)** Unknown protein. All Locus ID numbers are shown.Primers corresponding to all tested genes are listed in Supplementary Table S5. Values represent the mean ± SE of three biological replicates (*n* = 3).

To evaluate the impact of high levels of Fe on root gene expression profiles of the two rice cultivars, we used the Rice Gene Chip genome array (Affymetrix). Plants from EPAGRI 108 and BR-IRGA 409 cultivars were treated with excess Fe for 3 days. We chose this particular time point based on the photosynthetic activity data, which clearly indicated a slow but consistent increase in the photosynthetic capacity of EPAGRI 108 plants in day three after an initial drop upon exposure to excess Fe on days one and two (Figure 2), indicating that this could be the time point when tolerance-related traits started to be expressed. Exposure to excess Fe distinctly affected gene expression in the two cultivars: there were 423 up-regulated and 92 down-regulated genes in the susceptible cultivar BR-IRGA 409, while 43 up-regulated and 310 down-regulated genes were found in the tolerant cultivar EPAGRI 108 (Figure 6 and Supplementary Tables S1–S4). Strikingly, we found that there was no overlap between the responses in the two cultivars: 232 genes up-regulated in the susceptible cultivar were also down regulated in the tolerant one, while 29 down regulated in the susceptible were up regulated in the tolerant one. There were 191 and 15 exclusively up-regulated in the susceptible and tolerant cultivars, respectively; and 64 and 78 down regulated in the susceptible and tolerant cultivars, respectively (Figure 6). These results were confirmed by RT-qPCR analyses of 13 selected genes (Figure 7A–M). The microarray data was confirmed in most cases, with the exception of some genes that are regulated in opposite directions in the two cultivars, which did not show the expected down-regulation. This might be due to the fact that some genes shown as down-regulated in the microarray already have low expression under control conditions. Altogether, these results indicate that these cultivars respond very differently to excess Fe, and confirm the quality of our dataset.

**Figure 8:**
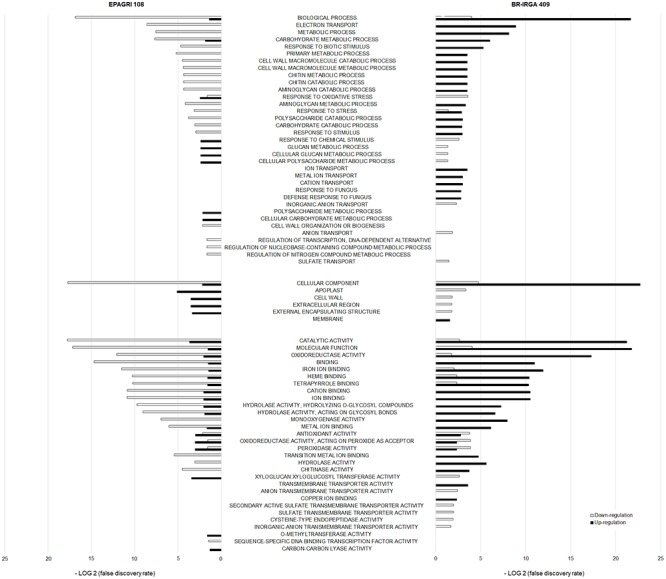
Comparison of Gene Ontology terms enrichment between the two cultivars up- and down regulated genes. Term enrichment is shown as a function of the False Discovery Rate observed. Terms enriched in up- or down-regulated gene sets of either BR-IRGA 409 or EPAGRI 108 are highlighted.

We conducted a Gene Ontology (GO) term enrichment analysis to have an overview of the cultivar-specific response to excess Fe (Figure 8). Data clearly showed that the two cultivars have very different enriched categories, and many of them are found in opposite regulation (i.e., up regulated in one cultivar, down regulated in the other). We found that “Ion Transport,” “Metal Transport,” and “Cation Transport” categories were enriched in the susceptible cultivar up regulated gene set, but not in the tolerant one. We also found many categories related to cell wall enriched in both cultivars. Interestingly, the tolerant cultivar has up regulated categories such as “Cell Wall,” “Apoplast,” “Extracellular Region,” which were all down regulated in the susceptible one. Moreover, the tolerant cultivar did not have categories associated with ion or metal transport up or down regulated (Figure 8).

In order to have a more in-depth, gene-by-gene view of the different responses of each cultivar, we analyzed the 15 top regulated genes up- and down-regulated (Table 3). We found among the top up-regulated genes in BR-IRGA 409 a vacuolar Fe transporter, OsVIT2, that is responsive to excess Fe (Zhang et al., 2012); an uncharacterized VIT-Like (VTL) transporter (69-fold), similar to the VTL proteins known to mediate Fe transport into vacuoles in Arabidopsis (Gollhofer et al., 2014); OsASR3 (abscisic stress ripening protein; 25-fold), which was already been shown to be responsive to drought stress (Joo et al., 2013), and aluminum toxicity (Arenhart et al., 2013); and one abscisic acid responsive protein (18-fold). Moreover, the well-known Fe-excess responsive Ferritin genes OsFER1 and OsFER2 (Stein et al., 2009b; Ricachenevsky et al., 2010) were also up-regulated in BR-IRGA 409 (3.8- and 4.3 fold, respectively; Supplementary Table S1), indicating that these plants are responding as expected to high Fe concentrations.

**Table 3:** Top 15 up- and down regulated genes in each cultivar.

Locus TIGR	BR-IRGA 409	EPAGRI 108	Gene description
*Up-regulated in BR-IRGA 409*		
LOC_Os04g45510	89.22443	-12.244819	hypothetical protein
LOC_Os04g45520	69.78652	-6.165692	VIT-Like Protein
LOC_Os02g37380	54.848434	-37.811863	Expressed protein
LOC_Os10g37160	41.434082	-56.202168	Cytochrome P450 CY89A2
LOC_Os09g23300	37.27906	NDEG	OsVIT2
LOC_Os11g31540	27.535797	-38.461082	BRASSINOSTEROID INSENSITIVE 1-associated receptor kinase 1 precursor, putative, expressed
LOC_Os05g08830	27.033407	-15.521343	Hypothetical protein
LOC_Os03g16030	27.02408	NDEG	17.4 kDa class I heat shock protein 3
LOC_Os01g72900	25.64241	-10.956484	OsASR3 (abscisic stress ripening protein 3)
LOC_Os09g19820	22.898518	-6.1851354	Aminopeptidase-like protein
LOC_Os06g09870	20.326283	-9.836528	Glycine-rich cell wall structural protein 2
LOC_Os01g58960	19.996674	-5.3904	Cytochrome P450 94A1, putative, expressed
LOC_Os07g46920	19.222033	-11.117482	Sex determination protein tasselseed-2, putative, expressed
LOC_Os01g43750	19.13674	-5.59671	Cytochrome P450 72A1, putative, expressed
LOC_Os12g29400	18.903513	NDEG	ABA-responsive protein, putative, expressed
*Down-regulated in BR-IRGA 409*		
LOC_Os02g43410	-109.300575	NDEG	OsYSL15 (Fe3+-phytosiderophore transporter)
LOC_Os03g19436	-102.14942	NDEG	OsNAS1 (Nicotianamine synthase 1)
LOC_Os03g19420	-63.42101	NDEG	OsNAS2 (Nicotianamine synthase 2)
LOC_Os10g18870	-57.646217	25.752077	Dirigent-like protein
LOC_Os03g46470	-38.56981	12.099378	OsIRT1 (Fe2+ transporter)
LOC_Os01g45914	-30.128687	NDEG	IRON MAN 1
LOC_Os02g20360	-27.718102	NDEG	OsNAAT1 (Nicotianamine aminotransferase)
LOC_Os10g18820	-22.484005	33.883545	Dirigent-like protein
LOC_Os12g36840	-14.281752	NDEG	Pathogenesis-related protein 10
LOC_Os07g15460	-13.20834	NDEG	OsNRAMP1 (putative metal transporter)
LOC_Os11g15624	-12.335097	NDEG	Unknown protein
LOC_Os02g43370	-12.134548	NDEG	OsYSL2 (Fe3+-phytosiderophore transporter)
LOC_Os01g65110	-11.203648	NDEG	Proton-dependent oligopeptide transport (POT) family protein
LOC_Os11g05390	-10.996121	NDEG	Tetracycline transporter protein, putative, expressed
LOC_Os03g22010	-9.311682	5.7919073	OsPrx41 (peroxidase)
*Up-regulated in EPAGRI 108*		
**LOC_Os10g18820**	-**22**.**484005**	**33**.**883545**	**Dirigent-like protein**
**LOC_Os10g18870**	-**57**.**646217**	**25**.**752077**	**Dirigent-like protein**
LOC_Os06g16640	-3.6726174	13.423064	Carboxyl-terminal peptidase, putative, expressed
LOC_Os04g46810	-7.2157893	12.945946	Cortical cell-delineating protein precursor, putative, expressed
**LOC_Os03g46470**	-**38**.**56981**	**12**.**099378**	**OsIRT1 (Fe2+ transporter)**
LOC_Os10g18760	-8.9875965	11.241121	Jasmonate-induced protein, putative, expressed
LOC_Os02g37260	-3.2897632	10.861882	Expressed protein
LOC_Os01g73170	-6.2279987	8.349177	OsPrx20 (peroxidase)
LOC_Os03g09980	-8.079094	7.5308027	Sulfate transporter 1.2, putative, expressed
LOC_Os11g07770	-8.848223	7.3968105	Dirigent-like protein
LOC_Os05g28770	-7.0391817	6.0021596	Expressed protein
**LOC_Os03g22010**	-**9**.**311682**	**5**.**7919073**	**Peroxidase 2 precursor, putative, expressed**
LOC_Os06g48180	-7.122961	5.211064	Xyloglucan endotransglucosylase/hydrolase protein (OsXTH10, OsXTH11, OsXTH12, or OsXTH18)
LOC_Os09g31430	NDEG	4.727019	Non-cyanogenic beta-glucosidase precursor, putative, expressed
LOC_Os06g20150	-4.356904	4.563937	OsPrx78 (peroxidase)
*Down-regulated in EPAGRI 108*	
**LOC_Os10g37160**	**41**.**434082**	-**56**.**202168**	**Cytochrome P450 CY89A2**
**LOC_Os11g31540**	**27**.**535797**	-**38**.**461082**	**Brassinosteroid insensitive 1-associated receptor kinase 1**
**LOC_Os02g37380**	**54**.**848434**	-**37**.**811863**	**Expressed protein**
LOC_Os11g46000	5.0010676	-30.422861	Von willebrand factor type A domain containing protein
LOC_Os02g36110	12.113743	-21.564749	Cytochrome P450 76C2, putative, expressed
LOC_Os04g10160	8.430319	-18.699465	Cytochrome P450 CYP99A1
LOC_Os04g09920	7.923875	-17.830105	Cytochrome P450 CYP99A1, putative, expressed
LOC_Os01g13610	7.5534124	-17.769463	Isoflavone reductase homolog IRL, putative, expressed
LOC_Os06g35700	6.695192	-17.551943	Reticuline oxidase precursor, putative, expressed
LOC_Os07g44440	13.513653	-16.21314	Peroxiredoxin, putative, expressed
**LOC_Os05g08830**	**27**.**033407**	-**15**.**521343**	**Hypothetical protein**
LOC_Os04g49210	11.567054	-14.981616	Naringenin,2-oxoglutarate 3-dioxygenase, putative, expressed
LOC_Os07g46846	3.5318127	-14.938069	Sex determination protein tasselseed-2, putative, expressed
LOC_Os07g23410	5.9368644	-14.20258	Omega-6 fatty acid desaturase, endoplasmic reticulum isozyme 2, putative, expressed
LOC_Os12g36830	6.6913185	-13.807509	Pathogenesis-related protein 10, putative, expressed

*Genes are shown in four blocks (BR-IRGA 409 up-regulated, BR-IRGA 409 down-regulated, EPAGRI 108 up-regulated, EPAGRI 108 down-regulated). Genes that are differentially expressed in both cultivars among the top 15 regulated genes appear in bold. Fold change in both cultivars is also shown (NDEG means Not Differentially Expressed in that cultivar)*.

Conversely, we found several genes related to Fe deficiency responses down-regulated in the susceptible cultivar when exposed to excess Fe (Table 3 and Supplementary Table S2). Among the top 15 down-regulated genes in BR-IRGA 409, eight genes are well-described as being up-regulated under Fe deficiency (Zheng et al., 2009; Grillet et al., 2018), including the Fe(III)-Deoxymugineic acid (DMA) transporter OsYSL15 (-109-fold) and the Fe(II)-Nicotianamine (NA) transporter OsYSL2 (-12-fold); the Fe^2+^ transporters OsIRT1 (-38-fold), and OsNRAMP1 (-13-fold); the DMA biosynthesis-related genes OsNAS1 (-102-fold), OsNAS2 (-63-fold), and OsNAAT1 (-27-fold); and the regulatory peptide OsIMA1 (IRON MAN 1; Grillet et al., 2018). Other genes that are commonly associated with the Fe deficiency regulon were also down regulated, but not among the top 15 genes, such as OsZIFL4/TOM1 (-6.2-fold; Nozoye et al., 2011; Ricachenevsky et al., 2011), OsYSL16 (-3.6-fold; Kakei et al., 2012), and OsMIR (-3.2-fold; Ishimaru et al., 2009). Thus, these results show that BR-IRGA 409 plants are down-regulating Fe acquisition-related genes.

In contrast, the tolerant cultivar EPAGRI 108 showed three dirigent (DIR)-like proteins among the top 15 up-regulated genes (7-, 25-, and 33- fold; Table 3 and Supplementary Table S3), which are related to lignin and lignin biosynthesis (Paniagua et al., 2017); two cell-wall modifying enzymes (Xyloglucan endotransglucosylase/hydrolase protein, 5/2-fold; a beta-glucosidase, 4.7-fold); and three peroxidases (8.4-, 5.7-, and 4.5-fold), which were already linked to metal stress (Kidwai et al., 2019). Interestingly, we found OsIRT1 up-regulated in this cultivar (12-fold), which is the opposite regulation found in the susceptible cultivar BR-IRGA 409. However, the absolute expression levels were extremely low, as shown in RT-qPCR data (Figure 7), and might not be physiologically relevant.

Among the top 15 genes down-regulated in EPAGRI 108 cultivar, the three most strongly down-regulated genes are also among the top 15 up-regulated genes in BR-IRGA 409, again demonstrating a clear opposite transcriptional regulation in these two cultivars upon excess Fe. Several Cytochrome P450 genes were also down-regulated (Table 3).

### Roots of the Tolerant Cultivar Show Increased Lignin Deposition Upon Excess Fe Treatment Compared to the Susceptible One

**Figure 9:**
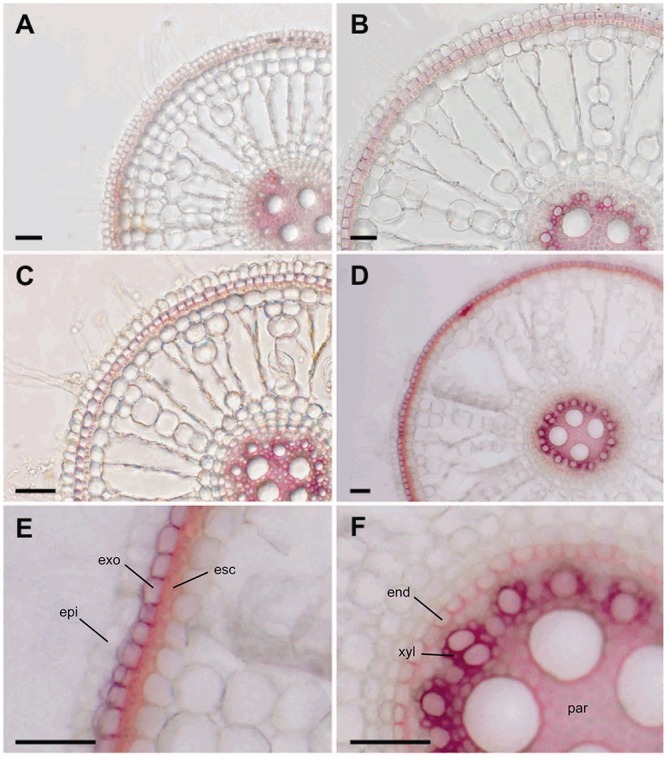
Root lignification under iron excess. Cross-sections of root sectors from BR-IRGA 409 **(A,B)** and EPAGRI 108 **(C,D)** plants maintained for 15 days under control **(A,C)** or iron excess **(B,D)** treatments, showing the extent of lignification (cell walls stained in red) indicated by the Wiesner reaction (acidified phloroglucinol solution). Magnifications from figure **D** show more intense cell wall lignification of exodermis (exo) and esclerenchyma (esc), in the outer layers of the cortex **(E)**, and lignin deposition in endodermis (end), xylem parenchyma (xyl), and pith parenchyma (par) cell walls **(F)**. Scale bars = 100 μm.

Based on the microarray data, we hypothesized that the tolerant cultivar might show tolerance to Fe toxicity because of cell wall modifications. Since DIR proteins are involved in lignin biosynthesis, we analyzed lignin deposition in roots of both cultivars under control and excess Fe conditions. We found almost no change in lignin deposition pattern in the susceptible cultivar BR-IRGA 409 when comparing root sections of plants under control (Figure 9A) and excess Fe (Figure 9B) treatments. However, root sections of plants from the tolerant cultivar EPAGRI 108 cultivated under control (Figure 9C) and excess Fe (Figure 9D) presented markedly distinct lignin localization.

Lignin deposition in roots of the tolerant cultivar increased both in the outer layers of the cortex (exodermis and sclerenchyma ring, Figure 9E), in u-shaped secondary cell wall deposits of the tertiary endodermis around the vascular cylinder, in the primary xylem parenchyma cells (intense red staining), and in the pith parenchyma cells (Figure 9F). These results indicate that altered lignin deposition might be involved in the tolerance mechanism found in EPAGRI 108.

### Lignin Concentrations in Roots of Susceptible and Tolerant Genotypes

**Figure 10:**
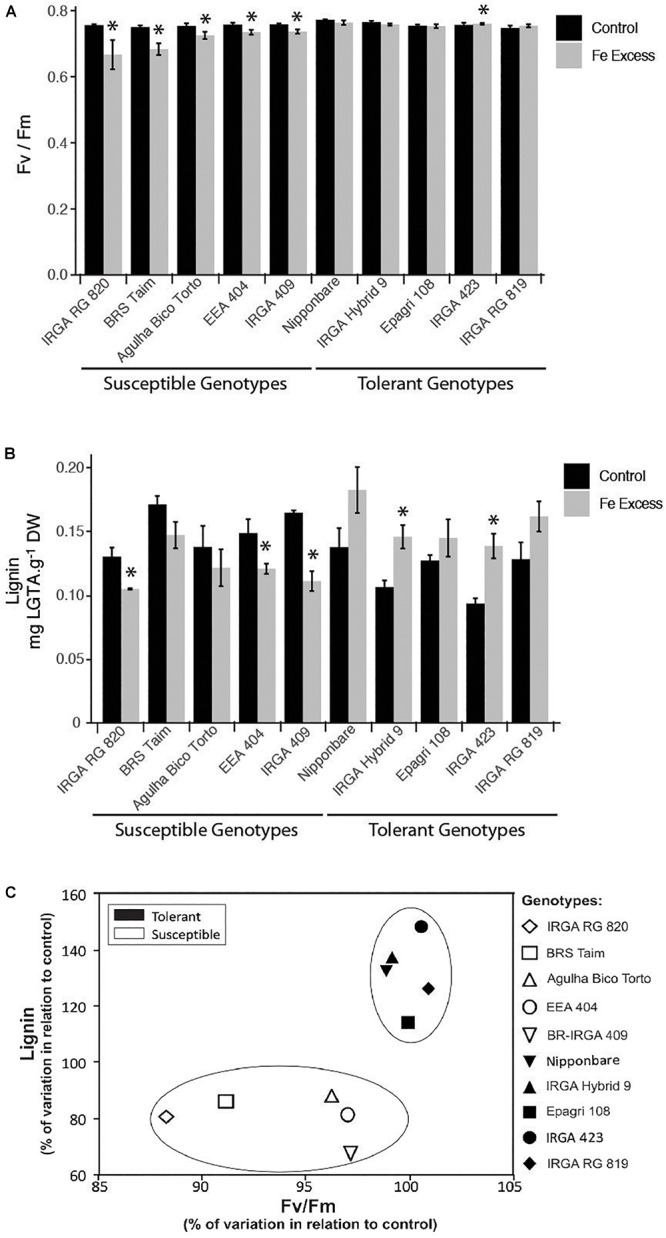
Effects of iron excess in plants from ten rice genotypes exposed to 500 mg L^-1^ FeSO_4_ (Fe+) or 6.5 mg L^-1^ FeSO_4_ (Ctrl) for 15 days: maximum photochemical efficiency (F_V_/F_M_) from leaves **(A)**; lignin concentration from roots **(B)**; and genotype distribution plot **(C)**, based on % variations observed in iron excess treated plants in relation to control plants (% variation in leaf F_V_/F_M_ values against % variation in root lignin concentrations). In **A** and **B**, the asterisks indicate means that are significantly different from control by the Student’s *t*-test (*P* ≤ 0.05). Experiments were performed using *n* = 8 to 9 plants per genotype.

Based on the clear change in lignin deposition, we decided to analyze the lignin concentration in other rice cultivars that might contrast in their Fe tolerance. In order to access Fe tolerance/susceptibility, we used maximum photochemical efficiency (F_V_/F_M_) values. Our susceptible and tolerant reference cultivars showed markedly different responses to excess Fe: BR-IRGA 409 showed a clear decrease in F_V_/F_M_ value upon excess Fe exposure, whereas EPAGRI 108 showed no difference compared to control plants (Figure 10A). Lignin quantification showed that the susceptible cultivar has a decreased in lignin concentration, while the tolerant one showed a slight increase (Figure 10B). A set of eight genotypes were also evaluated, and cultivars that showed decreased F_V_/F_M_ values when treated with excessive Fe were considered susceptible, whereas those showing no change (or slight increase in one case) were considered tolerant (Figure 10A). Clearly, tolerant cultivars showed increased lignin concentration in roots when plants were treated with excess Fe (Figure 10B). Conversely, susceptible cultivars showed decreased lignin (Figure 10B). Data from all ten cultivars were plotted comparing the % of variation in F_V_/F_M_ values and lignin concentration when exposed to excess Fe compared to the same cultivar under control conditions. Tolerant and susceptible genotypes grouped separately (Figure 10C), indicating that changes in total lignin concentrations are involved in tolerance to excess Fe.

## Discussion

### Contrasting Physiological Impacts of Excess Fe on the Two Rice Cultivars

Excess Fe led to decreased biomass accumulation (in shoots and roots), loss of chlorophyll and decreased photosynthetic activity in the susceptible BR-IRGA 409 cultivar (Figure 1–3). The tolerant cultivar, however, showed no change in biomass or chlorophyll, and slight decrease in photosynthetic parameters, which were mostly recovered during the course of the 9-day excess Fe treatment (Figure 2, 3). Previous work with the same rice cultivars showed that exposure to excess Fe did not indirectly induce deficiency of other nutrients, indicating a direct effect of excess Fe, a result that was later confirmed by field experiments (Silveira et al., 2007; Stein et al., 2009a). The toxic effects were directly related to accumulation of higher levels of Fe in shoots and roots of BR-IRGA 409 plants. Consistently, Olaleye et al. (2001) reported that high concentrations of Fe resulted in decreased plant weight and grain yield, and the growth retardation observed was not attributed to deficiencies of other nutrients, but to physiological problems directly originating from excessive Fe accumulation. Thus, our data confirmed the tolerant and the susceptible traits of these cultivars, validating the experimental approach used in this work.

In this study, photosynthetic activity decreased in plants from both cultivars after the initial period of exposure to excess Fe. However, the tolerant cultivar (EPAGRI 108) was able to fully recover its photosynthetic capacity after 6 days, a reasonable lag period for the induction of its tolerance mechanisms. For this reason, our transcriptomic analyses were performed at 3 days of exposure to excess Fe. Using A/Ci and light response curves, we could identify the impact of excess Fe on carbon fixation, affecting the maximum carboxylation rate (Vc_max_), electron transport rate (J_max_), and maximum apparent quantum yield (ϕ_m_) in the susceptible cultivar, but not in the tolerant one (Table 2). The reduction in Vc_max_ may result from the reduced rate of electron transport, limiting the amount of available energy for Calvin cycle enzymes. The reduction in chlorophyll concentration associated to lower electron transport rates may indicate a direct effect of excess Fe on the photosynthetic electron transport chain components (either LHCII or Cytb_6_/f). The toxic effects of Fe in photosynthesis were also observed in *Nicotiana plumbaginifolia* cuttings accompanied by photoinhibition, increased reduction of PSII and higher thylakoid energization (Kampfenkel et al., 1995). Excessive amounts of Fe in thylakoid membranes in the form of cytochrome b_6_/f complex showed correlation with photodamage to PSII, derived from excessive production of singlet oxygen in pea plants (Suh et al., 2002).

Data from independent studies showed that BR-IRGA 409 increases photorespiration under excess Fe (Pereira et al., 2013), and together with electron transport rate and carboxylation efficiency were proposed as good parameters to access rice Fe tolerance (Müller et al., 2017). Our tolerant cultivar showed no change in V_cmax_, J_max_ (Table 2), and Fv/Fm (Figure 9), in contrast with the susceptible one, which corroborate the use of photosynthetic parameters for easy, non-destructive access to Fe tolerance in rice genotypes (Pereira et al., 2013; Müller et al., 2017).

### Mechanisms of Fe Tolerance in the Two Rice Cultivars

Tolerance to Fe toxicity in rice can have three general possible sources of tolerance: Fe exclusion (i.e., avoiding Fe entering plant tissues); Fe uptake and further compartmentalization (i.e., in Fe storage proteins such as Ferritins, or in vacuoles); and Fe uptake associated with increased tolerance to the consequent reactive oxygen species using anti-oxidants (Wu et al., 2014). Of course, one given genotype can combine two or more tolerance mechanisms. Here both cultivars showed comparable concentrations of Fe in shoots and roots under control conditions, whereas exposure to excess Fe induced Fe accumulation in roots and shoots of both cultivars (Table 1). The susceptible cultivar, however, showed higher levels of Fe compared to the tolerant one on both organs, suggesting that the tolerant genotype is able to exclude Fe. Exclusion by roots can be achieved by increased oxidation capacity, resulting in higher Fe^2+^ oxidation to Fe^3+^, Fe precipitation and Fe plaque formation, limiting the absorption of Fe by the root system (Wu et al., 2014). After 9 days of excess Fe treatment, both cultivars showed increased Fe concentrations in the Fe plaque (Table 1). However, the susceptible cultivar (BR-IRGA 409) showed significantly higher levels of Fe in the Fe plaque than the tolerant cultivar (Table 1). These data suggest that the tolerant cultivar does not have increased oxidation power in roots, which would lead to higher levels of Fe in the apoplast. In fact, EPAGRI 108 decreased levels of Fe in the Fe plaque might be due to increased lignification, which would decrease root oxygen loss and therefore reduce root oxidation power. Thus, these data indicate that EPAGRI 108 tolerance is based at least partially on a distinct mechanism.

The increased Fe concentration in shoots of plants from the tolerant cultivar treated with excess Fe accumulation was significant, but with little effect on the plant phenotype compared to the susceptible one (Figure 1). Thus, it is likely that EPAGRI 108 also has Fe shoot – based tolerance as well. This is consistent with the observation that the susceptible cultivar has increased markers for ROS stress, while the tolerant one showed no difference compared to controls (Figure 4). Enzymatic detoxification of ROS could be involved in Fe toxicity responses (Fang et al., 2001; Majerus et al., 2007). APX activity, which could be part of a ROS tolerance, shoot-based mechanism as previously proposed (Wu et al., 2017), is induced only in the susceptible cultivar (Figure 5B), but not in the tolerant. Catalase activity was shown to be slightly higher in the tolerant cultivar (Figure 5A). Previous analyses of Fe tolerant and susceptible rice cultivars associated lower dehydroascorbate reductase, higher ascorbate oxidase activity and high rates of ascorbate reduction to tolerance (Wu et al., 2017), which were not tested in our experiments. Thus, EPAGRI 108 should be further investigated to identify the nature of its shoot-based tolerance mechanism.

### Fe Homeostasis-Related Genes Are Strongly Regulated in the Susceptible Cultivar

Our data clearly shows that the susceptible BR-IRGA 409 cultivar is responding as expected to Fe toxicity (Table 3 and Supplementary Tables S1–S5). Genes such as OsVIT2, OsFER1, and OsFER2 are up-regulated indicating that plants are compartmentalizing excessive Fe into vacuoles and ferritin holoproteins (Stein et al., 2009b; Zhang et al., 2012). Another interesting finding is that a putative, uncharacterized VTL transporter was one of the highly expressed genes under excess Fe. VTL were shown to transport Fe into the vacuoles in Arabidopsis (Gollhofer et al., 2014), indicating that the rice homolog might be important for Fe detoxification in vacuoles. Moreover, we found two ASR genes, OsASR1 and OsASR3, up-regulated in this cultivar. Both genes are up regulated by aluminum (Al) stress, and OsASR1 is involved in regulating the Al stress response in rice, acting as a transcription factor (Arenhart et al., 2013). Fe and Al stresses are common in acidic soils, and thus ASR proteins might be involved to some extent in Fe toxicity gene regulation as well. Alternatively, Fe and Al stress responses in roots might be convergent, since both result in changes in root length due to decreased elongation. This, however, remains to be tested.

As already described in other studies, the Fe deficiency responsive genes were down regulated under excess Fe, but exclusively in the susceptible cultivar. Fe uptake transporters OsYSL15, OsIRT1, and OsNRAMP1; nicotianamine and phytosiderophore synthesis enzymes (NAS and NAAT); and phytosiderophore secretion transporter OsZIFL4/TOM1 were among them (Table 3 and Supplementary Table S2). These indicate that plants from the susceptible cultivar are attempting to avoid Fe uptake under excess Fe, and is in agreement with previous observations (Quinet et al., 2012; Bashir et al., 2014). An Fe and Cu transporter, OsYSL16, was also found as down-regulated (Supplementary Table S2). OsYSL16 was shown to be important for Fe distribution in rice plants, and its activation-tagging (which results in higher steady-state expression levels) results in increased Fe efficiency (Kakei et al., 2012; Lee et al., 2012). Thus, OsYSL16 decreased expression under excess Fe might be also contributing to avoid Fe accumulation.

Interestingly, we also found the recently described OsIMA1 (LOC_Os01g45914; IRON MAN 1), a small peptide that was clearly involved in regulating the Fe deficiency response in Arabidopsis (Grillet et al., 2018). Microarray data already demonstrated that OsIMA1 was up regulated by Fe deficiency (Zheng et al., 2009) and heterologous expression in Arabidopsis showed it is able to regulate Fe deficiency responses (Grillet et al., 2018). However, OsIMA1 physiological role is not established yet. Our data shows that it may be also important to regulate rice responses to Fe toxicity, likely by down regulating the Fe uptake machinery. In agreement with that, OsMIR, an orphan rice small protein that is also involved in Fe deficiency responses (Ishimaru et al., 2009), was also down regulated in BR-IRGA 409, again indicating the importance of such small regulatory peptides in regulating Fe concentration in plants.

However, it is puzzling that we haven’t found any commonly regulated genes in the tolerant cultivar compared to the susceptible one. We considered that 3 days of Fe excess treatment would be a good time point to identify contrasting differences in the two cultivars, based on physiological data. However, we should consider that sampling at later time points, when Fe would build up to higher levels in the tolerant cultivar, could show us similar genes regulated in EPAGRI 108 as observed in the susceptible BR-IRGA 409 after 3 days treatment with Fe excess. A time course transcriptional experiment could reveal the dynamics of this regulation, and how tolerant and susceptible cultivars may differ in the timing at which some genes are up and down regulated. Thus, our non-overlapping expression patterns would likely not be maintained throughout Fe excess responses for more than 3 days.

### Root Lignification Might Be Involved in Tolerance to Excess Fe

As inferred from the distinct Fe accumulation and distribution in the plant body between the two studied cultivars, the capacity to accumulate lower levels of Fe greatly contributes to the tolerance character of EPAGRI 108 plants. Based on the gene expression pattern and on the root anatomical analysis described in this work, root cell wall lignification and remodeling could play an important role in providing such a capacity to EPAGRI 108 plants. The tolerant cultivar showed only 42 genes up-regulated by excess Fe after 3 days of exposure. Among these, we found four peroxidases, one laccase and three dirigent proteins, most of them among the top up-regulated genes (Table 3 and Supplementary Table S3) indicating that the dirigent-guided lignin deposition might be up-regulated in the roots of the tolerant cultivar under excess Fe. The synthesis of coniferyl alcohols by peroxidases and laccases is known to lack region and stereoselectivity (Paniagua et al., 2017). Dirigent proteins stipulate, at regional and stereochemical levels, the outcome from the coupling of two molecules of *E*-coniferyl alcohol to produce the lignan (+)-pinoresinol (Davin et al., 1997). One characterized dirigent protein was shown to require the provision of one-electron oxidation through an auxiliary source, such as laccases, peroxidases and monooxygenases for its activity, and to be expressed mainly in lignifying tissues (Burlat et al., 2001). Two enzymes involved in lignin biosynthesis, Caffeoyl-CoA *O*-methyltransferase and *O*-methyltransferase ZRP4 (Raes et al., 2003; Boerjan et al., 2003), were also up-regulated in EPAGRI 108 (Supplementary Table S3). These results indicated that lignin deposition might be involved in Fe tolerance observed in EPAGRI 108.

Our data further corroborated this hypothesis showing that other Fe tolerant cultivars induce lignin deposition in roots, whereas Fe susceptible cultivars decrease total lignin (Figure 10). A recent work showed that heterologous expression in Arabidopsis of OsPRX38, a rice Class III peroxidase, leads to higher tolerance to arsenic (Kidwai et al., 2019). Interestingly, expression of OsPRX38 caused increased expression lignin biosynthesis genes such as Caffeoyl-CoA *O*-methyltransferase, which results in root lignification, providing an apoplastic barrier for arsenic diffusion and therefore less arsenic uptake by Arabidopsis plants (Kidwai et al., 2019). Similarly, expression of Arabidopsis AtHMA4 (a membrane-localized Zn/Cd efflux transporter) in tobacco resulted in decreased Cd concentration in roots and shoots. Lignin biosynthesis genes were up-regulated when these plants were exposed to Cd, with consequent increased lignification, which blocked apoplastic diffusion of Cd (Siemianowski et al., 2014). Cu excess or Mn excess were also shown to induce lignification in Arabidopsis and rice, respectively (Lequeux et al., 2010; Dziwornu et al., 2018). Our data shows for the first time that lignin biosynthesis is linked to excess Fe responses and to Fe tolerance.

Lignin deposition increased both in the exodermis and in the endodermis, possibly limiting Fe uptake into the root and Fe translocation to the shoot, respectively. The exodermis and the endodermis are the outer- and innermost cortical layers of a root, and serve as filtration sites for the passive movement of ions between the soil solution and the stele (Ma and Peterson, 2003). These are the root tissue layers where rice deposits Casparian strips, which act as apoplastic diffusional barriers (Cai et al., 2011). Interestingly, work in Arabidopsis showed that Casparian strips are entirely made of a lignin polymer without suberin, contrary to what was previously accepted (Naseer et al., 2012). The Casparian strip works as a barrier for nutrient diffusion both into and out of the stele, which can have consequences for root to shoot translocation of minerals (Doblas et al., 2017; Ricachenevsky et al., 2018). The transcriptional control and further steps for its formation are being dissected in detail. Among these, a dirigent protein named ENHANCED SUBERIN 1 (ESB1)/AtDIR10 was shown as essential for Casparian strip integrity in Arabidopsis (Hosmani et al., 2013). However, rice orthologous proteins to the ones involved in Casparian strip synthesis and maintenance are not among the differentially expressed genes in our datasets. Therefore, lignin deposition in response to excess Fe in the tolerant cultivar is performed by a different set of genes, likely reinforcing the Casparian strip barrier or adding other layers of lignification in similar tissues to avoid Fe diffusion into the roots. Based on our data (Figure 10) this new mechanism might be common to other rice cultivars, indicating a new avenue for rice Fe tolerance breeding and engineering.

In EPAGRI 108 roots under iron excess, the additional lignification of cell walls of the xylem parenchyma cells, which surround the tracheal metaxylem elements, may represent an extra barrier against iron translocation. Parenchyma xylem cells are the final water transport route before the tracheal cell entrance, after passage through the epidermis and cortical layers (including exoderm and endoderm). Therefore, an increase in lignification in the parenchyma of the xylem can function as a final “line of defense” against Fe transport (another apoplastic barrier). In the roots of *Brachiaria decumbens*, all xylem cells (i.e., including xylem parenchyma cells) are thickened with an evident deposition of lignin on the wall because of the increased concentration of heavy metals (Gomes et al., 2011).

## Conclusion

Our results described two rice cultivars, one tolerant and one susceptible, to excess Fe. We provide datasets for gene expression of two contrasting rice cultivars regarding Fe toxicity tolerance under early Fe excess stress. We suggest that tolerance in the EPAGRI 108 cultivar, and likely in other rice germplasm, could be linked to increased cell wall lignification in the root cortex layers (endodermis and exodermis), as well as in the central cylinder (primary xylem and pith parenchyma cell), which would be a new mechanism for rice tolerance to high Fe concentrations. The data provided here could be important for further studying the responses of rice cultivars to Fe excess, and uncovering a possible Fe tolerance mechanism linked to cell wall remodeling.

## Materials and Methods

### Plant Material, Growth, and Treatments

Seeds from rice (*Oryza sativa* L. ssp. *indica*) genotypes used in this work were provided by the Rice Breeding Group from IRGA (Instituto Rio Grandense do Arroz, Brazil). BR-IRGA 409, IRGA RG 820, BRS Taim, Agulha Bico Torto, EEA 404, Nipponbare, IRGA Hybrid 9, EPAGRI 108, IRGA 423, and IRGA RG 819 are part of the germplasm bank of IRGA, and are commonly used for testing different traits in breeding programs of the Institute. The two genotypes used in most experiments are cultivated in southern Brazil and were previously characterized as tolerant (EPAGRI 108) and susceptible (BR-IRGA 409) to Fe toxicity (Silveira et al., 2007). Seeds were surface sterilized in 70% ethanol for 2 min, followed by 1.5% NaClO_4_ for 1 min, and then washed with abundant distilled water and germinated on moistened filter paper in Petri dishes. The seedlings were kept in the dark during the first 48 h, transferred to 16 h/8 h day/light regime (65 μmol m^-2^s^-1^ of photosynthetic active radiation) at 28°C for 2 days and then transferred to pots with vermiculite, watered with nutrient solution consisted of 1.42 mM NH_4_NO_3_; 0.4 mM NaH_2_PO_4_; 0.5 mM K_2_SO_4_; 1.7 mM CaCl_2_.2 H_2_O; 1.7 mM MgSO_4_.7H_2_O; 9.5 μM MnCl_2_.4 H_2_O; 0.07 μM (NH_4_)_6_Mo_7_O_24_.4H_2_O; 20 μM H_3_BO_3_; 0.16 μM ZnSO_4_.7H_2_O; 0.16 μM CuSO_4_.5H_2_O; 35.6 μM FeCl_3_.6H_2_O, and 0.07 mM citric acid (Yoshida et al., 1976). After 10 days, plants were transferred to hydroponic conditions, using the same nutrient solution, and after 10 more days plants were subjected to excess Fe (500 mg L^-1^ of iron) or control concentration (6.5 mg L^-1^), both using FeSO_4_ as the Fe source. To avoid possible effects of sulfur concentrations, Na_2_SO_4_ was added to the control solution to obtain equimolar sulfur concentration in both treatments. To maintain the concentration and keep Fe soluble, nutrient solutions were replaced every 72 h.

### Dry Weight and Chlorophyll Determinations

After 9 days of treatment, plants were separated in shoots and roots, immediately frozen in liquid nitrogen and kept at –20°C until further analysis. Shoots and roots were dried at 60°C to constant weight for the determination of DW. Fully expanded leaves were ground in liquid nitrogen and chlorophyll extracted in acetone 85%. Total chlorophyll (chlorophyll *a*+ chlorophyll *b*) was quantified by measuring absorbance at 663 nm and 645 nm, and the concentrations calculated as in Stein et al. (2009a).

### Gas Exchange Measurements

Gas exchange measurements were performed after 1, 2, 3, 6, and 9 days of exposure to the Fe treatments, using a portable photosynthesis system (LI-6400, LiCor Inc., Lincoln, NE, United States). All determinations of photosynthetic rate were performed using a reference CO_2_ concentration of 400 μL L^-1^, 1000 μmol m^-2^ s^-1^ photosynthetic photon flux (PPF) and leaf temperature of 22°C, using only the youngest fully expanded leaf. Light response curves were performed using a reference CO_2_ concentration of 400 μL L^-1^, leaf temperature of 22°C and a PPF range from 1500 to 0 μmol m^-2^s^-1^. CO_2_ response curves were performed using 1000 μmol m^-2^ s^-1^ PPF, leaf temperature of 22°C, with reference CO_2_ concentrations ranging from 800 to 50 μL L^-1^. All photosynthetic response curves were performed using only the youngest fully expanded leaf, after 6 days of exposure of plants to treatments (control and excess Fe), and the photosynthetic parameters were estimated according to the biochemical model described in Farquhar et al. (1980).

### Fe Concentration in Shoots, Roots, and Fe Plaque

To determine the Fe concentration in the Fe plaque, root systems were washed in abundant distilled water and immediately incubated for 3 h in cold dithionite-citrate-bicarbonate solution (Taylor and Crowder, 1983) and the Fe concentration determined by atomic absorption spectrometry (Varian-Model Spectra 10/20, Victoria, VIC, Australia). After extraction of Fe plaque, root systems were washed in distilled water and dried at 60°C. Dry samples (shoots and roots) were ashed at 500°C for 3 h. Ashes were digested with concentrated HCl and Fe was quantified by atomic absorption spectrometry.

### Oxidative Damage to Lipids, Proteins, and H_2_O_2_ Determination

Lipid peroxides were extracted in 80% ethanol from fully expanded leaves and lipid peroxidation determined by measuring the concentration of TBARS as described by Hodges et al. (1999). Oxidative damage to proteins was determined by the quantification of carbonyl groups, by derivatization with 2,4-dinitrophenyl-hydrazine. Fully expanded leaves were ground in cold extraction buffer [50 mM Tris (pH 8.0), 2 mM EDTA, 1 mM phenylmethylsulfonyl fluoride and 1 mM benzamidine], centrifuged at 12,000 × *g* for 15 min at 4°C, and the supernatants immediately used for carbonyl determination according to Levine et al. (1990) and normalized with the protein concentration determined using the dye-binding method (Bradford, 1976). H_2_O_2_ was quantified spectrophotometrically (Cintra 5, GBC Scientific Equipment, Victoria, VIC, Australia) after extraction with 0.1% trichloroacetic acid and reaction with KI in the dark (Alexieva et al., 2001). The amount of H_2_O_2_ was calculated using a standard curve prepared with known concentrations.

### Antioxidant Enzymes Activity

For all enzymatic activity determinations, fully expanded leaves were ground in cold extraction buffer containing 50 mM of sodium phosphate buffer (pH 7.4), 1% (w/v) polyvinylpyrrolidone, 1 mM EDTA, 1 mM phenylmethylsulfonyl fluoride, and 1 mM benzamidine. The homogenate was centrifuged at 12,000 × *g* for 15 min at 4°C and the supernatant immediately used for enzymatic assays.

Ascorbate peroxidase activity was determined according to Klapheck et al. (1990), from the decrease in absorbance at 290 nm; CAT activity was determined following the decrease of absorbance at 240 nm due to H_2_O_2_ consumption (Cakmak and Marschner, 1992); and SOD activity was quantified as described by Beyer and Fridovich (1987), using 15 min of illumination and recording the absorbance at 560 nm. All enzymatic assays were performed at 25°C as initial activities, with no lag period, and total protein concentration determined by the dye-binding method (Bradford, 1976).

### Global Gene Expression Analysis and Real-Time PCR

For global gene expression analysis, highly purified total RNA was obtained using NucleoSpin RNA II (Macherey-Nagel) from roots of EPAGRI 108 and BR-IRGA 409 plants exposed for 3 days to control or excess Fe conditions. RNA purity and quality was assessed by absorbance measurement (260 and 280 nm ratio) and by analysis on a Bioanalyzer (Agilent Technologies). Global gene expressions analyses were performed using the GeneChip Rice Genome Array (Affymetrix), which contains probes to query 51,279 transcripts representing two rice subspecies, with approximately 48,564 transcripts from *japonica* and 1,260 transcripts from the *indica* subspecies, respectively. 5 μg of total RNA were used as starting material for each sample, which were labeled using the one-cycle target labeling and control reagents (Affymetrix). Target preparation, micro-chip hybridization, washing, staining, and scanning were carried out according to manufacturer’s instructions (Affymetrix). The Affymetrix GeneChip Operating Software 1.2.1 was used for washing and scanning in the Fluidics Station 450 (Affymetrix) and the Scanner 3300 (Affymetrix), respectively. Sample quality was assessed by examining the 3′ to 5′ intensity ratios of the Poly-A and hybridization controls, and the housekeeping genes. For further data analysis, the probe intensity files (.cel) were imported into the ArrayAssist software (Stratagene) and normalization and probe summarization were performed using the Robust Multichip Analysis algorithm (Irizarry et al., 2003), followed by variance stabilization. Variance stabilization was performed to suppress noise by addition of a fixed quantity to all linear scale signal values.

To identify differentially expressed genes, a Student’s *t*-test (using *P* ≤ 0.05) was performed and the genes that showed significant differences and were up- or down-regulated by threefold or more were considered to be differentially expressed. Averages from three biological replicates for each sample were used for analysis.

The results from the microarray analysis were validated by real-time PCR. Total RNA was isolated from roots of rice plants exposed for 3 days to control or excess Fe treatments, and one microgram was treated with DNAse and reverse transcribed. Polymerase chain reactions were performed in a StepOne real time PCR System (Applied Biosystems), using SYBR^®^ Green (Invitrogen) to monitor dsDNA synthesis. The following standard thermal profile was used for all PCRs: 95°C for 5 min; 40 cycles of 95°C for 15 s, 60°C for 10 s, 72°C for 15 s, and 60°C for 35 s. Relative expression was determined based on Schmittgen and Livak (2008) method with slight modifications, as described previously (Dametto et al., 2015). Primer sequences corresponding to all tested genes are listed in Supplementary Table S5. The microarray data obtained is publicly available at Gene Expression Omnibus^1^ under the accession number GSE131287.

### Gene Ontology Analyses and Venn Diagram

Gene Ontology analyses were performed using PlantGSEA^2^. The 20 most highly enriched categories were selected, and showed with false discovery rate transformed to Log_10_ base. Categories were separated in “Biological Process,” “Cellular Component,” and “Molecular Function.” The Venn diagram was generated using Venny^3^.

### Histochemical Staining

Plants were harvested after 15 days of treatment. The five millimeters most distal from the root tip were harvested from each root and fixed in 1.5 mL of 2.5% (w/v) glutaraldehyde and 3% (w/v) formaldehyde in phosphate buffer (pH 7.2). Hand-cut transverse sections were taken from fixed roots, after phosphate buffer (pH 7.2) washing followed by rinse in distilled water. The presence of lignin was determined by the Wiesner reaction (Wiesner, 1878), which gives a characteristic red coloration due to cinnamic aldehydes contained in lignified cell walls (Pomar et al., 2002), when viewed under bright field light microscopy. Sections of the roots were soaked in 1:1 aqueous solution of 2% (w/v) phloroglucinol and 70% (v/v) HCl, transferred to a glass-slide, topped with one drop of glycerin and covered with a cover slip. Observations and images were made using a Leica DM light microscope equipped with a Leica DFC500 Digital Camera system.

### Chlorophyll *a* Fluorescence

Chlorophyll fluorescence was evaluated using a portable Chlorophyll Fluorometer (OS-30p Opti-Sciences Inc., Hudson, NH, United States). The fast kinetics rising transient (OJIP) measurements were made on attached youngest fully expanded leaves, which were dark-adapted for 12 h. For these measurements, eight plants from each genotype and treatment were used. The F_V_/F_M_ ratio or trapping probability, TR0/ABS, which is the probability that an absorbed photon will be trapped by the Photosystem II reaction center with the resultant reduction of Q_A_ (primary electron acceptor of Photosystem II) was estimated from the JIP test parameters (Strasser and Strasser, 1995; Krüger et al., 1997). Experiments were performed using *n* = 8 to 9 plants per genotype.

### Lignin Quantification

After 15 days of treatment, roots of nine plants from each genotype were harvested and dried in an oven (60°C, 9 days). The eight millimeters most distal from the root tip were harvested from each root for lignin analysis. Root lignin concentration was determined from the protein-free cell wall fraction by the formation and quantification of lignin-thioglycolic acid (LTGA) (Santos et al., 2004, modified proportionally to use 0.05 g of dry roots per sample). The LTGA pellets were dried at 60°C, dissolved in 0.5 M NaOH and diluted to yield an appropriate absorbance for spectrophotometric determination at 280 nm. For lignin’s standard curve preparation, different concentrations from 0.005 to 0.5 mg ml^-1^ of lignin (Aldrich 37,096-7 Lignin, alkali, 2-hydroxy-propyl ether) were prepared in the same way (reaction with thioglycolic acid and HCl) and the absorbance at 280 nm was determined. Lignin concentration was expressed as milligram LTGA per gram of dry weight. Experiments were performed using *n* = 8 to 9 plants per genotype.

### Statistical Analysis

Means from most physiological and biochemical analyses were compared by ANOVA, followed by Duncan test, according to Quinn and Keough (2002). F_V_/F_M_ and lignin concentration means were compared to the respective controls by the Student’s *t*-test (P ≤ 0.05) using the SPSS Base 12.0 for Windows (SPSS Inc., Chicago, IL, United States). Differences were considered significant when *P* ≤ 0.05.

## Data Availability

All datasets for this study are included in the manuscript and the Supplementary Files. The microarray data obtained in this work is available at Gene Expression Omnibus^4^ under the accession number GSE131287.

## Author Contributions

RJS and JPF conceived the study. RJS, GLD, LS, and MGS conducted the experiments. RJS, AdAJ, FKR, LMGR, NITZ, RS, and JPF analyzed the data. JPF, LMGR, NITZ, and RJS provided the experimental tools. RJS, FKR, and JPF wrote the manuscript. All authors approved the current version of the manuscript.

## Conflict of Interest Statement

The authors declare that the research was conducted in the absence of any commercial or financial relationships that could be construed as a potential conflict of interest.

## Footnotes

1 https://www.ncbi.nlm.nih.gov/geo/

2 http://structuralbiology.cau.edu.cn/PlantGSEA/analysis.php

3 http://bioinfogp.cnb.csic.es/tools/venny/

4 https://www.ncbi.nlm.nih.gov/geo/
